# Explaining the increase in coronary heart disease mortality in Syria between 1996 and 2006

**DOI:** 10.1186/1471-2458-12-754

**Published:** 2012-09-09

**Authors:** Samer Rastam, Radwan AL Ali, Wasim Maziak, Fawaz Mzayek, Fouad M Fouad, Martin O'Flaherty, Simon Capewell

**Affiliations:** 1 Syrian Centre for Tobacco Studies, Aleppo, Syria; 2 Robert Stempel College of Public Health and Social Work, Florida International University, Miami, FL, USA; 3 University of Memphis, School of Public Health, Division of Epidemiology and Biostatistics, Memphis, TN, USA; 4 Department of Public Health and Policy, University of Liverpool, Liverpool, UK; 5 Syrian Center for Tobacco Studies Syrian Society against Cancer building, St.Aleppo, Shihan, Syria

**Keywords:** Coronary heart disease, Mortality, Modelling

## Abstract

**Background:**

Despite advances made in treating coronary heart disease (CHD), mortality due to CHD in Syria has been increasing for the past two decades. This study aims to assess CHD mortality trends in Syria between 1996 and 2006 and to investigate the main factors associated with them.

**Methods:**

The IMPACT model was used to analyze CHD mortality trends in Syria based on numbers of CHD patients, utilization of specific treatments, trends in major cardiovascular risk factors in apparently healthy persons and CHD patients. Data sources for the IMPACT model included official statistics, published and unpublished surveys, data from neighboring countries, expert opinions, and randomized trials and meta-analyses.

**Results:**

Between 1996 and 2006, CHD mortality rate in Syria increased by 64%, which translates into 6370 excess CHD deaths in 2006 as compared to the number expected had the 1996 baseline rate held constant. Using the IMPACT model, it was estimated that increases in cardiovascular risk factors could explain approximately 5140 (81%) of the CHD deaths, while some 2145 deaths were prevented or postponed by medical and surgical treatments for CHD.

**Conclusion:**

Most of the recent increase in CHD mortality in Syria is attributable to increases in major cardiovascular risk factors. Treatments for CHD were able to prevent about a quarter of excess CHD deaths, despite suboptimal implementation. These findings stress the importance of population-based primary prevention strategies targeting major risk factors for CHD, as well as policies aimed at improving access and adherence to modern treatments of CHD.

## Background

Coronary heart disease (CHD) is projected to be the leading global cause of death and disability by 2020 [1]. Most of the global burden from cardiovascular disease (CVD) morbidity and mortality is taking place in the developing countries, and CVD has already overtaken infectious diseases as the main threat to public health [2-5]. In the Eastern Mediterranean Region (EMR), CVD mortality accounts for almost a third of all deaths, and is mainly due to CHD [2]. According to available evidence, mortality rates from CHD are on the rise for most countries of the EMR [6,7]. In Syria, one of the low-middle income countries of the EMR (population ≈ 20 million; 2006), CHD is the main cause of CVD mortality, and together with stroke accounts for about half of all-cause mortality [8]. One of the notable patterns of CHD morbidity and mortality in the EMR compared to developed countries is their earlier onset, which translates into loss of productive years and more strain on the already burdened livelihood of poor communities [9].

Designing effective interventions to reduce CHD burden in developing countries needs to be guided by local data to identify main risk factors driving CHD incidence, and the most effective approach to reduce CHD burden considering the resources available [10]. Unfortunately, for most of the developing world, including countries of the EMR, very few reliable data exist about the main drivers of CHD mortality in terms of treatment and risk factors. As part of a new project funded by the EU (Medchamps: Mediterranean Studies of Cardiovascular disease and Hyperglycaemia: Analytical Modelling of Population Socio-economic transitions), we started collecting and analysing such data for several countries in the Mediterranean region (Syria, Tunisia, Palestinian authority, and Turkey).

According to WHO estimates, CHD mortality in Syria showed increasing trends during the past two decades [11,12]. This study aims to explain these trends between 1996 and 2006, and to examine factors associated with them, using a validated modelling approach.

## Methods

The IMPACT mortality model was adopted in this study to quantify the effects on CHD mortality attributable to changes in each population risk factors and treatment modalities between 1996 and 2006. The IMPACT model was previously validated in developed and developing countries such as New Zealand, China and Scotland [13-15]. Briefly, the IMPACT model is used to estimate the number of coronary heart disease (CHD) deaths prevented by each specific cardiac intervention, or risk factor decline or vice versa. The comparison between the increase of treatment for CHD disease and cardiovascular mortality represents another approach applied to obtain a more precise estimate of the role of the reduction of each risk factor. In this study, adults’ data including: (1) number of CHD patients, (2) use of specific medical and surgical treatments, (3) effectiveness of specific treatments for CHD, (4) population trends of major cardiovascular risk factors (smoking, total cholesterol, hypertension, obesity, and diabetes), were incorporated in the model. Details are shown in Additional file 1.

### Data sources and assessment

Syrian data on risk factors and current practices for treating CHD patients were identified through extensive search of published and unpublished data and complemented with specifically designed surveys. All data sources were critically appraised by Syrian Centre for Tobacco Studies (SCTS) and Medchamps teams and the results of this process are presented in the Additional file 1.

Data used to populate the model included: a) population statistics and CHD related mortality, b) patient numbers in specific CHD groups (Myocardial Infarction (MI), Congestive Heart Failure (CHF), Chronic Angina Pectoris (AP), c) use of specific medical and surgical treatments for CHD, and d) population trends in major cardiovascular risk factors. The main outcome of the model was number of deaths prevented or postponed (DPP) due to changes in risk factors or the application of certain treatments.

#### Information on the population demographic changes

Demographic information for Syria between 1996–2006 were obtained from the Syrian bureau of Statistics [16]. Numbers were comparable to the numbers provided by the U.N. department of economics and social affairs [17]. Numbers of CHD related deaths for both years were obtained from the WHO Global Health Observatory [11]. These numbers were cross-validated with data provided by the Aleppo Household Survey (AHS), which was conducted in 2004, and in which mortality estimates were calculated from participant-reported deaths among their adult household members (>20 years) during the five years preceding the time of the survey. [18]. Because mortality data for Syria are not available for every year, mortality rates for 1996 were estimated from data of 1985 and 2004, which are the closest available data points to 1996 using the geometric mean of the age-specific death rates [19]. These data were obtained from the WHO Statistical Information System [11,12].

#### Data on population risk factors’ trends

Data for the year 2006 were obtained from two epidemiological studies. The first one was the STEPwise survey – conducted by WHO in the rural and urban areas of Syria in 2003 with people up to 65 years were included; and in which a nationally representative sample of 9184 participants were surveyed [20]. The second one was the Aleppo Diabetes Survey (ADS) – conducted in 2006 –in which a representative sample of 1168 aged ≥ 25 years from the city of Aleppo (2^nd^ largest city with population ≈ 2.5 million) were surveyed [21]. Syrian data for the year 1996 were not available; therefore, they were extrapolated from the Palestinian Authority data, because those data were the most complete and standardized ones among neighbouring countries and because of the ethnic and cultural similarities of the two populations.

#### Data on CHD hospital admissions and (in-hospital) treatment

Because no valid information was available on CHD hospitalization and treatment, the SCTS has conducted a survey in 2009 specifically for this project. The survey covered 6 major hospitals. Three of these hospitals provide cardiac care in the Aleppo province, and the other 3 were general hospitals.

#### Data on treatment use for CHD in the community

Data for 2008 were obtained from an outpatient survey conducted by SCTS in 2009 using a random sample of five health centres. Information on treatment uptake in the community was verified with the help of experts’ opinion using a special questionnaire about treatment of CHD, assuming that treatment use did not change significantly between 2006 and 2008.

#### Data on efficacy of therapeutic interventions

Data were based on the results of recent meta-analyses and randomised controlled trials [22-52]. The Mant and Hicks approach was used to correct for polypharmacy in individual patients [53].

Population and mortality data were obtained from openly available sources; data on current risk factor profile and treatment uptakes were obtained from surveys conducted by SCTS; while data on risk factor profile for 1996 with permission to be used was obtained with from Institute of Community and Public Health, Birzeit University.

### Assessment of trends in CHD deaths (1996–2006) and the relative contribution of treatment and risk factors

First, the number of CHD deaths *expected* in 2006 was calculated by indirect age standardisation based on the assumption that 1996 mortality rates had persisted unchanged until 2006. The number of CHD deaths actually *observed* in 2006 was then subtracted. The difference between the two represents the rise in CHD deaths that the model needed to explain.

The number of additional deaths attributable to changes in cardiovascular risk factors was estimated using regression coefficients (β) to quantify the population mortality impact of changes in risk factors measured as continuous variables (blood pressure, total cholesterol and BMI). For categorical risk factors, including diabetes, physical inactivity and smoking, the population attributable risk fraction was used based on the standard formula:

$$\text{PAR}=\frac{\text{Prevalence x}\left(\text{Relative Risk}-1\right)}{\left[\text{Prevalence x}\left(\text{Relative Risk}-1\right)+1\right]}$$

To assess the effect of the treatment type on CHD mortality, the model needed to include all medical and surgical treatments in 1996 (the base year) and 2006 (the final year). However, detailed information on treatment modalities was not available for the year 1996. The data included in the model for 1996 were therefore estimated based on feedback from the ten leading local cardiologists in Aleppo, who treated CHD patients in hospitals and the community at that time using a short survey with standardized questions commonly used in the IMPACT model.

The mortality reduction attributable to each treatment type was calculated as the number of patients in each age and sex group multiplied by three factors: 1) the baseline, age-specific case fatality rate observed in that group, 2) the relative mortality reduction reported in published meta-analyses [22-52], and 3) the proportion receiving that specific treatment ( Additional file 1). Case-fatality data were obtained from large, unselected, population-based patient cohorts [54]. Survival benefit over a one-year time interval was used for all treatments.

Potential overlaps between different groups of patients were identified and appropriate adjustments were made. Patient group calculations and all other assumptions are detailed in the Additional file 1. Adherence (defined as the proportion of treated patients actually taking therapeutically effective levels of the prescribed medication) was assumed to be 100% among hospital patients, 70% among all symptomatic community patients, and 50% among asymptomatic community patients, based on the literature. [55-57]

### Sensitivity analysis

Because of the uncertainties surrounding some of the values used in the modelling, multi-way sensitivity analysis using Brigg’s analysis of extremes was used*.*[58]

## Results

Between 1996 and 2006, CHD mortality rates in Syria increased by 64% (from 129 to 212 per 100,000 person-years; 58% in men and 75% in women). This resulted in 6370 additional CHD deaths in 2006 compared to the number expected had the 1996 mortality rates persisted. Mortality increases were seen in men and women in all age groups (Table 1).

**Table 1:** **Population sizes and death rates from CHD in Syria, 1996 and 2006**.

			**Men**				
Age groups	**25-34**	**35-44**	**45-54**	**55-64**	**65-74**	**+75**	**+25**
Population in 1996	1063649	652453	381580	269255	171321	62074	2600332
Population in 2006	1478983	1053893	636889	351702	213778	107432	3842677
Deaths in 1996 (number)	379	542	522	743	1423	583	4191
Deaths in 2006 (number)	955	1573	1263	1327	3000	1674	9793
**Death rates per 100,000 in 1996**	36	83	137	276	831	939	161
**Death rates per 100,000 in 2006**	65	149	198	377	1403	1558	255
% Change (crude)	81%	80%	45%	37%	69%	66%	58%
	**Women**						
Age groups	**25-34**	**35-44**	**45-54**	**55-64**	**65-74**	**+75**	**+25**
Population in 1996	1019177	634984	381124	271637	168367	66709	2541998
Population in 2006	1432147	1016763	627629	363840	234341	127094	3801814
Deaths in 1996 (number)	145	195	256	314	911	642	2463
Deaths in 2006 (number)	315	517	680	604	2268	2061	6445
**Death rates per 100,000 in 1996**	14	31	67	116	541	963	97
**Death rates per 100,000 in 2006**	22	51	108	166	968	1622	170
% Change (crude)	55%	66%	61%	44%	79%	68%	75%

### Major CHD risk factors

Of the 6370 excess CHD deaths in 2006, 5140 (minimum 3165 and maximum 7705) can be explained by changes of major cardiovascular risk factors between 1996 and 2006. This represents 80.7% of the observed increase in CHD- mortality.

Changes in risk factors were complex: 0.31 mmol/L rise in total cholesterol (0.22 mmol/L in men and 0.39 mmol/L in women), 9 mmHg rise in mean systolic blood pressure (SBP) (6 mmHg in men and 11 mmHg in women), 4.7% rise in diabetes prevalence (4.1% in men, 5.3% in women), and a 1.8 units rise in BMI (1.5 in men, 2.2 in women).

Approximately 52% (2680, minimum 1620 and maximum 4110) of the excess deaths that were explained by changes in cardiovascular risk factors were attributable to increases in SBP*;* 19% (1000, 640 and 1460) to increases in total cholesterol*;* 15% (770, 490 and 1105) to increases in diabetes prevalence*;* 9% (470, 270 and 710) to increases in BMI*;* and 4% (225, 145 and 320) to increases in smoking prevalence*.* (Table 2).

**Table 2:** Deaths attributable to population risk factor changes in Syria between 1996 and 2006

RISK FACTORS^*^	**Risk factor levels 1996 2006**		**Relative Change (%) in risk factor 1996-2006**	**Relative Risk (RR**^**1**^**) (or βeta Coefficient)**^**2**^	**Increase in Deaths**			
					**Best Estimate**	**Minimum estimate**	**Maximum estimate**	**Proportion of overall deaths**
**Cholesterol ***mmol/l*					**1000**	**635**	**1460**	**15.7%**
*Cholesterol (men)*	*5.01*	*5.23*	*4.5*	β 0.65				
*Cholesterol (women)*	*5.07*	*5.46*	*7.6*	β 0.65				
**Smoking (***% )*				1,60 (1–9 cig/d);	**225**	**145**	**320**	**3.5%**
				1.80 (10–19 cig/d);				
				2.10 (>20 cig/d)				
*(% men smoking)*	*55.3*	*58.6*	*6.0*					
*(% women smoking)*	*10.0*	*15.0*	*49.4*					
**BMI***Kg/m*^*2*^					**470**	**270**	**710**	**7.3%**
*BMI (men)*	*26.75*	*28.26*	*5.6*	β 0.02				
*BMI (women)*	*29.08*	*31.24*	*7.4*	β 0.02				
**Diabetes (**%)					**770**	**490**	**1105**	**12.0%**
*Diabetes (men)*	*7.4*	*11.5*	*55.6*	2				
*Diabetes (women)*	*10.1*	*15.5*	*52.6*	2				
**Population systolic BP** mm/Hg					**2680**	**1620**	**4110**	**42.1%**
*Population BP (men)*	*125.6*	*131.8*	*4.9*	β 0.053				
*Population BP (women)*	*118.7*	*129.9*	*9.5*	β 0.053				
**Physical inactivity**	*-*	*-*	*-*	-	**-**	-	-	**-**
**Estimated total risk factor effects**					**5140**	**3155**	**7705**	**80.7%**

^*^ Numbers of increases in deaths were rounded to nearest 0 or 5.

### Medical and surgical treatments

**Figure 1:**
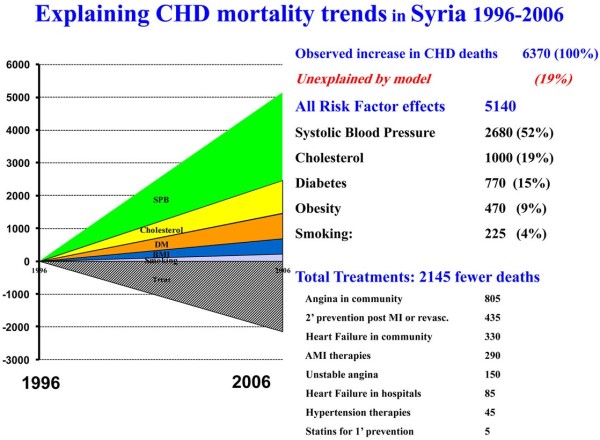
Coronary heart disease deaths prevented or postponed by treatment and risk factors changes in the Syrian population between 1996 and 2006.

Medical and surgical treatments together prevented or postponed approximately 2145 deaths in 2006 (minimum 1135, maximum 5475). In the absence of these treatments, there would have been approximately 2145 additional CHD deaths. The total treatment effect represented a 34% reduction of the overall CHD mortality (minimum estimate 18% and maximum 86%, Figure 1). Treatment of angina pectoris in the community explained over 38% of reduction in CHD deaths (805, minimum 665 and maximum 2125), most being attributable to aspirin and statin therapies (Table 3).

**Table 3:** Deaths prevented or postponed by medical and surgical treatments in Syria in 2006

**TREATMENTS**	**Patients eligible**	**Treatment uptake (%)**	**CHD deaths prevented or postponed**			**Proportion of overall deaths**
			**Best Estimate**	***Minimum estimate***	***Maximum estimate***	**Prevented or postponed (%)**
**Acute myocardial infarction**	**18002**		**285**	**120**	**540**	**4.5%**
* Hospital resuscitation*		*0.05*	*13*	*8*	*23*	*0.2%*
* Aspirin alone*		*0.96*	*151*	*62*	*266*	*2.4%*
* Thrombolytic alone*		*0.59*	*128*	*53*	*266*	*2.0%*
* Beta blockers*		*0.82*	*29*	*12*	*59*	*0.5%*
* ACE inhibitors*		*0.74*	*56*	*23*	*115*	*0.9%*
* Treatments in 1996 subtracted*			*−92*	*−37*	*−191*	
**Secondary Prevention post infarction**	**78976**		**372**	**125**	**949**	**5.8%**
* Aspirin*		*0.46*	*112*	*37*	*278*	*1.8%*
* Beta blockers*		*0.47*	*125*	*41*	*310*	*2.0%*
* ACE inhibitors*		*0.33*	*92*	*30*	*230*	*1.4%*
* Statins*		*0.28*	*90*	*29*	*223*	*1.4%*
* Warfarin*		*0.04*	*16*	*5*	*39*	*0.2%*
* Rehabilitation*		*0.10*	*36*	*12*	*89*	*0.6%*
* Treatments in 1996 subtracted*			*−97*	*−29*	*−219*	
**Secondary Prevention post revascularisation (5 years)**	**106517**		**65**	**22**	**164**	**1.0%**
**Angina**			**803**	**664**	**2124**	**12.6%**
* CABG surgery (1997–2006)*	*106517*	*0.10*	*97*	*-*	*-*	*1.5%*
* Aspirin*	*319974*	*0.44*	*511*	*168*	*1273*	*8.0%*
* Statins*	*319974*	*0.34*	*443*	*116*	*1322*	*6.9%*
* Treatments in 1996 subtracted*			*−248*	*−75*	*−671*	
**Treatments for unstable angina**	**22877**		**150**	**92**	**403**	**2.3%**
**Heart Failure with Hospital admission**	**6927**		**84**	**25**	**223**	**1.3%**
* ACE inhibitors*		*0.57*	*41*	*11*	*122*	*0.6%*
* Beta blockers*		*0.22*	*22*	*6*	*67*	*0.4%*
* Spironolactone*		*0.49*	*64*	*21*	*160*	*1.0%*
* Aspirin*		*0.80*	*55*	*18*	*135*	*0.9%*
* Treatments in 1996 subtracted*			*−99*	*−30*	*−262*	
**Heart failure in the community**	**52251**		**332**	**104**	**854**	**5.2%**
* ACE inhibitors*			*112*	*29*	*327*	*1.8%*
* Beta blockers*			*108*	*35*	*269*	*1.7%*
* Spironolactone*			*226*	*74*	*563*	*3.6%*
* Aspirin*			*97*	*32*	*240*	*1.5%*
* Treatments in 1996 subtracted*			*−211*	*−66*	*−546*	
**Hypertension Treatments**	**863425**		**45**	**8**	**311**	**0.7%**
**Statins for Primary Prevention**	**307295**		**6**	**2**	**65**	**0.5%**
Total treatment effects*			**2145**	**1165**	**5630**	**33.7%**

* Numbers of category totals of deaths prevented or postponed were rounded to nearest 0 or 5, totals may therefore not always sum exactly.

ACE, angiotensin-converting enzyme; CABG, coronary artery bypass graft surgery.

Secondary prevention following acute myocardial infarction explained over 17% of reduction in CHD deaths (370)*,* mostly due to beta blockers and aspirin). Approximately 15% (330) of deaths were prevented by heart failure treatments in the community (particularly spironolactone), and 14% (290) were attributable to initial treatments for acute myocardial infarction (especially thrombolysis and aspirin). However, the mortality reductions attributable to coronary surgery and statins for primary prevention were modest (4.7% and 2.3% respectively, Table 3).

### Model validation and fit

The model explained approximately 81% of the total CHD mortality increase in the Syrian population between 1996 and 2006. The remaining 19% was unexplained and might reflect data limitations or other unmeasured factors such as physical inactivity, stress dietary factors.

It is perfectly reasonable to instead present the total deaths to explain as 8,515 deaths (6,370 + 2,145) deaths. In which case the 5140 deaths attributable to risk factor changes would represent approximately 60% of the total mortality increase.

The relative contributions of the risk factors studied remained relatively consistent, irrespective of whether the best, the minimum, or the maximum DPP estimates were used.

## Discussion

Coronary heart disease mortality increased in Syria by over 60% between 1996 and 2006. These trends resembled those in other developing countries such as China (using the same IMPACT model), as well as India [14,59]. Much of this mortality rise can be attributed to adverse trends in major risk factors such as blood pressure, cholesterol and diabetes. This is the first study to examine factors underlying the rise of CHD mortality in Syria. It, therefore, provides important and timely information that could guide national efforts to reduce the burden of CHD in Syria. It also can potentially provide useful information for other comparable Arab countries in the EMR.

The largest fractions of CHD excess mortality paralleled the increases in major CHD risk factors, with almost 60% of the mortality increase being attributable to elevated blood pressure and total cholesterol. These trends are consistent with the overall shift in Arab societies towards urbanization, modernization and lifestyles characterized by unhealthy diet and inactivity. Available data from Syria and other Arab countries in the EMR consistently show dramatic increases in obesity and diabetes, whereby overweight and obesity among adults in the EMR range from 25% to a staggering 82% [60]. In Syria obesity now affects over half (52%) of adult women [21]. These unfavourable trends contribute to an adverse cardiovascular risk factors’ profile, including a higher prevalence of diabetes and elevated cholesterol levels, as is seen in this study. The common behavioural roots underlying CVD risk increases in the Syrian population potentially highlight opportunities to reduce mortality rates through population–based interventions promoting healthy eating and active lifestyles. This also emphasizes the need for a national strategy to address non-communicable diseases in Syria using a combination of health promotion, fiscal measures, market controls and community involvement to encourage healthy lifestyles [61]. The contribution of smoking to increased CHD mortality was modest, as smoking prevalence hardly changed between 1996 and 2006.

This reflects a lack of effective tobacco control policies in Syria, and a complex political situation, where the government agency responsible for tobacco production and sale is much more powerful that the tobacco control unit at the ministry of health.

Modern medical treatments together prevented or postponed 2145 excess deaths in Syria in 2006, equivalent to about one fourth of the total CHD mortality. The biggest contributions came from long-term management of CHD, mainly for secondary prevention, angina, and heart failure. The contributions from surgery and angioplasty were small, accounting for 4% of the mortality reduction. Our previous research in Syria showed that in spite of high prevalence of CHD risk factors, such as hypertension, awareness and, consequently, treatment and control of such conditions tended to be low [21]. For example, our research has shown that about 40.6% of population aged 18–65 years had hypertension, only 11.8% of them were aware of it, and only 8.6% were under treatment [8]. For diabetes, which affects 15.6% of population aged 25 years and above in Syria, only 11.2% were diagnosed, and only 16.7% of treated cases had their diabetes controlled adequately [62]. This modest contribution of medical treatment to reducing CHD mortality points out to a great potential for improvement in this area.

Treatment uptakes were moderate (60%-90%) for relatively cheap drugs, such as aspirin, thrombolytic therapy, and beta blockers, but much lower for more expensive surgical procedures. There was essentially no utilization of coronary artery bypass graft (CABG), primary transcutaneous coronary angioplasty (PTCA), rehabilitation, nor cardiac pulmonary resuscitation (CPR) in the community. In our study, surgical procedures had a very limited role in avoiding CHD deaths, resulting in approximately 100 fewer deaths (≈ 5% of total treatment benefit). This small role may reflect the effects of the high cost of these procedures, the lack of access to specialized care for the general population, the limited availability of expertise and facilities needed to perform such procedures and the underdevelopment of the private and public health insurance sectors--a situation similar to that found in other developing countries, such as China [14]. This contrasts with the higher uptake in developed countries where, for instance, community resuscitation (i.e. out of health care facilities) reached 48% in England and Wales [63], and 50% in New Zealand [13].

The IMPACT modelling in this study used a comprehensive approach, synthesizing data about all major risk factors and all standard treatment options used in the Syrian population between 1996 and 2006. However, this approach also has some limitations. The data used were of variable completeness and quality. We critically appraised the available data to ensure adequate quality for our modeling. For example, we did not rely on national death registry data for causes of death because these were incomplete and of questionable quality, instead, preferring mortality data from WHO estimates [11,12]. In addition, when data were not available, we used explicit assumptions including data from a comparable neighboring (Palestinian) population.

It is also perfectly reasonable to present the total deaths to explain as 8,515 deaths (6,370 + 2,145). In which case the 5140 deaths attributable to risk factor changes would represent approximately 60% of the total mortality increase.

Lag times were not formally considered in the model. However, increasing evidence suggests that substantial mortality increases or falls may occur within a few years of risk factor changes [64]. Furthermore, certain assumptions were needed to fill in the gaps for missing information. For example, for the small group aged 65–74 years where risk factor information was not available we had to make assumptions based on the risk factors levels in the nearest age groups. These assumptions are detailed in the Additional file 1 and were supported by local expert opinions and literature from the region. All were tested in the sensitivity analysis. However, none of these limitations are expected to influence the validity of our main results, as the IMPACT model used in this study was developed to deal with such data problems.

## Conclusions

CHD mortality in Syria rose by about 60% between 1996 and 2006. More than two-thirds of this rise was attributable to changes in major risk factors such as blood pressure and total cholesterol. Despite suboptimal use, CHD treatments prevented or delayed 2145 CHD deaths in 2006 compared to 1996. These results emphasise the importance of advocating adequate, early, and continuous treatments for CHD within the community.

Risk factor changes in the general population are important drivers of the recent CHD mortality rises, and mainly reflect lifestyles characterised by unhealthy eating habits and physical inactivity. These should be the priority targets for national efforts to reduce cardiovascular morbidity and mortality. This will represent great challenges, because effective, population-based policies will require clear public health vision, multi-sectoral approaches, adequate resources and firm political commitment [64].

## Competing interests

The authors declare that they have no competing interest.

## Authors' contributions

SR originated the research question, conducted the analyses, interpreted the findings, and wrote the first dart of the article. RA contributed to the acquisition of data, analysis and interpretation of findings, and drafting of the manuscript. WM contributed to the interpretation of the findings, assisted with the editing of the article and supervision of the study. FM assisted with the editing of the article. FMF contributed to the acquisition of data. MOF contributed to analysis and interpretation of findings. SC contributed to drafting of the manuscript, analysis, interpretation of findings, critical revision, and supervision of the study. All authors read and approved the final manuscript.

## Pre-publication history

The pre-publication history for this paper can be accessed here:

http://www.biomedcentral.com/1471-2458/12/754/prepub

## Supplementary Material

Additional file 1 The caption for the additional file is: Syria Impact Model; Data Sources Assumptions and Risks. Click here for file
