# Supplementary material for: Explaining the increase in coronary heart disease mortality in Syria between 1996 and 2006
Source: BMC Public Health. 2012 Sep 9;12:754. doi: 10.1186/1471-2458-12-754 (PMC3485167; doi:10.1186/1471-2458-12-754)
Supplement: Additional file 1 — The caption for the additional file is: Syria Impact Model; Data Sources Assumptions and Risks. [file 1471-2458-12-754-S1.doc]

**SYRIA IMPACT Model - Technical Appendix**

**Data sources, assumptions and risks**

**General Procedures to deal with data limitations**

Some our data come from different and not complete sources. Some lacked breakdown by age groups and gender. We therefore used – when required- some general procedures to overcome such problems:

1. A lot of data sources have 65 years as the upper age range; in these cases estimates for age groups above 65 was assumed to be equal to the last age group.
2. When we have a zero or very low or very high value for some age group, in such cases we estimate the parameter by smoothing, averaging the rate using the two adjacent age groups.
3. In cases when we have a very few number of events in a specific age group, we combined this age group with the adjacent age groups.
4. In some cases these estimations were informed by age-related trends in the adjacent groups.

**Population Data:**

We have three sources; all of them provide population structure by age and gender:

1. Syrian bureau of Statistics: they provide data up to 2009. No future prediction. The oldest group are people older than 65 years.
2. U.N. Department of economics and social affairs website. They have yearly population estimates from 1950 to 2010 and different projections up to 2050 (medium-fertility variant, high-fertility variant, low-fertility variant and constant-fertility variant).
3. **U.S. Census Bureau from their website (**[**www.census.gov**](http://www.census.gov/) **), they have population projections up to 2050, the older group is up to 100 years and older.**

We compared all sources for 2003-2009; they provided reassuringly similar age structures, and the difference between them being usually less than 10%,

**Mortality Data:**

We checked three sources of information:

1. Data from **death certificates and official health departments**: these data list only major categories of diseases without precise identification of the primary cause of death- so we have categories such as heart disease and so on.

However, these data seem to be incomplete, unreliable or inconsistent. As an example, Ministry of Health data say that 49.2% of all death cases are due to circulatory diseases, ([MOF](http://www.moh.gov.sy/en/Statistics/MorbidityMortality/tabid/342/Default.aspx)), compared to 30% of all death cases according to WHO mortality country fact sheet 2006 ([WHO](http://www.who.int/whosis/mort/profiles/mort_emro_syr_syrianarabrep.pdf))

1. We therefore used data from the **Aleppo Household Survey (AHS).**

This survey was a cross-sectional survey conducted in 2004 by SCTS. The target population of AHS was adults 18–65 years of age. AHS used a two stage, stratified and cluster sampling, with the target population divided into two strata, formal and informal zones. The total sample size was 2038. This survey contained data about:

- *Obesity:* defined as BMI >=30
- *Waterpipe and cigarette smoking*
- *Blood pressure and hypertension:* Hypertension was conventionally defined as systolic blood pressure equal or higher than 140 mmHg, or diastolic blood pressure equal or higher than 90 mmHg, or self-reported treatment for hypertension.
- *Mortality data:* in this survey mortality estimates were calculated on the basis of participant-reported deaths occurring in the past 5 years among their adult (>20 years) household members. Participants were asked to name the main condition leading to that death from a list of main causes of death.

The survey provides us with the *heart diseases mortality rates* breakdown by age and gender.

We then estimated the number death cases due to heart diseases using the population statistics and mortality rates.

1. WHO Global Health Observatory <http://apps.who.int/ghodata/>

Data include death estimates for all causes by major age groups and by gender. We compared our estimates with the estimates for cases of ‘Hypertensive heart disease’ and ‘Ischemic heart disease’, the general numbers are comparable; for example the estimated number for death cases due to IHD in the age group 15-59 years was 3,752 in our estimation vs. 3,073 in the WHO estimation.

1. For some age groups such as females aged 45-54 years, we used the smoothing techniques described above to overcome the zero number of events in this age group.
2. CHD deaths 1996:

We calculate the numbers as following:

- - First we used a detailed mortality profile for Syria at the W.H.O. website at <http://www.who.int/whosis/mort/download/en/index.html>. These data contain details age, gender and cause specific death number for 1985 in Syria. Using these numbers and total population at each age and gender age group we calculate the rates for CHD death among population.
  - Then we calculate the CHD death rates in 2006 using our data
  - We assumed linear trends: that mortality rates at 1996 for each gender and age group in 1996 are midway between the above two rates (1985 and 2006)
  - Then using the population profile at 1996 we estimated the CHD death cases at 1996 for each gender and age group

**Risk Factors:**

We used three sources of data:

1. **Aleppo diabetes survey-** This survey was a cross-sectional survey conducted in **2006** by SCTS. The target population in this survey was adults age >=25 years residing in Aleppo. A Two-stage cluster sampling was used with a total sample size of 1168. This was the main source of data we used, because we have the raw data and we can break it down by age groups and gender.

This survey collected data about:

- *Obesity:* defined as BMI >=30.
- *Waterpipe and cigarette smoking.*
- *Blood pressure and hypertension:* Hypertension was conventionally defined as systolic blood pressure equal or higher than 140 mmHg, or diastolic blood pressure equal or higher than 90 mmHg, or self-report treatment for hypertension.
- *Low physical activity* that was defined as no regular physical activity (less than once a week).
- *Blood glucose and diabetes:* Diabetes was defined as a reported history of physician-diagnosed type 2 diabetes, or a fasting plasma glucose (FPG) levels >= 126 mg that was measured during the survey.
- *Blood cholesterol and hypercholestermia* defined as having blood cholesterol >= 240 mg/dL (measured during the survey)
- *Blood triglycerides, LDL and HDL cholesterol* (measured during the survey).

For older age groups, we used the techniques described above.

1. **Stepwise survey:** This survey was conducted by MOF and WHO in 2003. A national representative sample of 9184 participants was selected from all regions of Syria. This survey contains data about *self report diabetes, & smoking*. Data were presented by gender and age groups with 10 years interval, starting from 20 years.
2. For old data (from the 1990s), we use the Palestine data: data was collected up to 65 years old, for older age groups we assume the same values as the adjacent age group (55-64 years).

We reviewed the data about physical activity, but decided to ignore it due to problems with different definitions. Based on key informant interviews, we assumed that there were no significant changes in physical activity during the last 10 years.

We also ignore data about possible trends in vegetable and fruit intake as much as we do not have consistent and reliable sources for 2006 and 1996

**Treatment uptakes**

These data are also collected mainly from 2 surveys:

1. **Heart Disease Survey:** This survey was conducted by SCTS in Aleppo. This survey covered the three major hospitals that provide cardiac care in Aleppo province. We selected a random sample of 10% of all patients’ records in **2008**. The sample size was in general (N=569) larger than the estimated sample size required to get 95% confidence interval with 5% alpha level.

- Data that were collected from patients’ records include:

1. Daily treatments: dose and frequency.
2. Discharge diagnosis.
3. Discharge treatment, dose and frequency
4. **Outpatient Survey conducted by SCTS**. In this survey a random representative sample of private and public health centers was selected (7 health centers), and in each center a random sample of 10% of the records were examined by a professional team. Data about *heart disease (angina pectoris, post MI, post CABG or PTCA, and heart failure), diabetes and stroke* were collected.

- Data that were collected from the records include:

1. Treatments: dose and frequency.
2. Data on primary prevention (statin use) was collected from the household surveys described above

We made the following assumptions in estimating treatment uptakes:

1. For some treatments (*statins for primary prevention, and antihypertensive medication*) we used data from the ***diabetes*** ***and Aleppo household*** *surveys* (described above), which include questions about antihypertensive and lipid lowering medications.
2. Sample size became relatively small when the sample was brokendown by age groups and gender. While this is a limitation, this limitation can not be ruled out as much as we have a low prevalence of cardiac diseases in some age groups such as heart failure in women 25-34 years.
3. For older age groups we used the above described techniques.
4. We did not have the data for secondary prevention after MI for previous years (1995-2005). So we calculated the percentages based of the following assumptions that were summarized after the discussions with key informants.
   1. Data for treatment uptake in 2006 was used as the base for estimation the percentages of treatment uptakes during the previous 10 years
   2. Based on expert opinions we assumed that statin use was 0% during 1995-1999, then it is usage increased linearly from 2000 up to 2006
   3. Based on expert opinions we assumed that aspirin, warfarin, and beta blockers usage did not change significantly during this period
   4. Based on expert opinions we assumed that ACI Inhibitors use was 0% in 1995, its usage increased linearly till 2006
5. CPR in hospitals for acute MI admitted to hospitals: expert opinions suggest 5%
6. AMI (EMERGENCY) admissions (STEMI) - Proportion of PTCA that are STEMI: 0.6: Based on expert opinions

**Patient numbers:**

**Hospital AMI:**

- - First, we calculated the number of AMI in our sample from the **Heart Disease survey**
  - Then, we estimated the total number of AMI in the three major hospitals based on the total number of heart patients and the total MI cases
  - Then, we estimated the total number of AMI in Aleppo city by assuming based on expert opinion that almost half of AMI cases are usually admitted to these three major heart hospitals.
  - Then we estimated the total number of AMI admitted to hospital in Syria using the census data about the population of Aleppo and of Syria
  - For people older than 85 years we combined this group with people aged 75-84

The total number of AMI admitted to hospitals is estimated to be 18,002; which means a rate of 101 AMI admission case per 100,000 per year, also this number is consistent with estimate of the total number of death cases due to ischemic heart disease (WHO Global Health Observatory) is 12,126

**Unstable angina admitted to hospitals**

We used the same method to estimate the number of unstable angina patients, which result in 22,605 cases and rate 126 per 100,000 per year

**Heart Failure necessitating at least one admission to hospital**

The same method was used also to calculate the number chronic heart failure admitted to hospitals with the using of the total beds (instead of total beds at intensive care units), but the estimation becomes very big (99,334 case)

This bias occurred because patients do not go randomly to any hospitals, and many seek an admission to the big heart hospitals. This means that we can use another approach and assume that most people actually admitted to cardiac hospitals and the real number of admission is 10 times less (about 6,845), which may be more realistic (compared to number of AMI cases). From the second point, patients with heart failure are usually admitted more frequently to hospitals, so the number of admissions is much higher than number of patients. Experts say that on average, a patient with CHF is usually admitted 2-3 times per years (most patients are poorly educated which leads to poor management)

In the model, we used the second scenario which result 6,845 CHF cases and a rate of about 38 per 100,000 per year; In conclusion, this is a good place to implement sensitivity analysis

**CPR and CABG uptake**

- Community CPR: considered to be 0% according to experts
- In-hospital CPR: considered to be 5% (expert opinions)
- Uptake of CABG after AMI: considered to be 0% according to experts

**Secondary prevention (past data)**

We used the following assumptions:

1. the base for the number is the number of hospitalized AMI in 2006
2. 10% fewer in each preceding year
3. 10% case fatality rate every year

**Secondary prevention following CABG/PTCA (10 years)**

1. We started the calculation for the year 2006 by multiplying number of patients with angina in the community by percentage of them who have CABG
2. For previous years we assumed that the numbers are 90% of the following year

**Angina in the community (Aspirin & Statin)**

- It is not clear if that means who take either aspirin or statin or both together. Based on expert opinion, everybody who takes statin also takes aspirin, so we used the statin data alone
- From the treatment data, we estimated the % of statin treatment of angina.

The number of patients with angina was calculated similar to heart failure in the community described later

**CABG and PTCA Survival:**

- First we estimated the number of PTCA and CABG in 2008 for Syria, using % of angina patient who was treated by CABG or PTCA and estimated number of angina patient’s (explained above)
- Second we compared the total number with the total number of CABG in 2008 by some hospitals and expert opinions and we found that they are rational
- Then we estimated a linear increase of CABG and PTCA from 1995 to 2008, this seems rational as much as number of operations is related mainly to the capacities in hospitals and not to the number of patients which is still a lot higher
- Then with an expert we put an estimated survival rate for each year and calculate the total survivals based on that

**Heart Failure in the community**

- - First, we used the **Heart Clinics Survey.** We excluded all not ischemic heart cases, then we classified IHD into the following categories (Angina, post CABG, post MI, and CHF). We assumed that the percentages of these diseases are fixed for the same age group and gender and reflect the situation in the community.
  - From the **Heart Survey,** we estimated the total percentages of heart diseases by age and gender up to 65, and from diabetes survey for more advance ages (comparing heed and diabetes surveys the proportion is in the range 0.67 to 1.83 (one group was
  - We used these percentages and the total population of Syria to estimate the number of these cases (including CHF) in the community.

Also this approach carry the possibilities of bias, because patients with CHF go tot the clinics more frequent than patients with other heart diseases, so percentages from clinics may not represent real percentages in the community.

**Uptake of Lipid-lowering drugs %**

- - We assumed it is about 50% (compliance) based on expert opinion.

**Antihypertensive medication – Numbers**

- - From Heed and Diabetes surveys we obtained antihypertensive medication uptake
  - Then we calculated the number based on population statistics

**Part I - Summary of the model**

**Table1: Summary of the model**

|  | **DPPs*** | **%** |
| --- | --- | --- |
| **Observed rise** | **6372** |  |
| **Risk Factors**  Smoking  Systolic Blood Pressure  Cholesterol  Physical Activity  BMI  Diabetes | **5770**  224  2737  1573  0  468  768 | **80.7%** |
| **Treatments** | **-2145** | **-33.7%** |

**DPPs*: Death prevented or postponed**

**Treatment Effects**

|  | **DPPs*** | **%** |
| --- | --- | --- |
| **Initial treatments for acute MI**  Hospital CPR  Thrombolysis  Aspirin  Beta blockers  ACE inhibitor  Minus DPPs in base year | **287**  13  128  151  29  56  -91 |  |
| **Unstable Angina**  Aspirin & heparin  Aspirin  Minus DPPs in base year | **150**  222  3  -76 |  |
| **Secondary prevention after MI**  Aspirin  Beta blockers  ACE inhibitors  Statins  Warfarin  Rehabilitation including exercise  Minus DPPs in base year | **372**  112  125  92  90  16  36  -97 |  |
| **Secondary prevention after CABG/PTCA:**  Aspirin  Beta blockers  ACE inhibitors  Statins  Warfarin  Rehabilitation  Minus DPPs in base year | **65**  25  35  9  34  1  6  -44 |  |
| **Angina in the community**  CABG surgery (1997-2007)  Aspirin  Statins  Minus DPPs in base year | **803**  97  511  443  -248 |  |
| **Heart Failure in the hospital**  ACE inhibitors  Beta blockers  Spironolactone  Aspirin  Minus DPPs in base year | **84**  41  22  64  55  -99 |  |
| **Heart Failure in the community**  ACE inhibitor  Beta blockers  Spironolactone  Aspirin  Minus DPPs in base year | **332**  112  108  226  97  -211 |  |
| **Primary Prevention**  Statin for primary prevention  Antihypertensive medications | **51**  6  45 |  |

**DPPs*: Death prevented or postponed**

**Part II – Population, CHD death and CHD mortality rate**

***Table 2: Population and CHD Death***

|  | **Population** | | **CHD Deaths** | | **CHD Mortality Rate**  **(per 100,000)** | | |
| --- | --- | --- | --- | --- | --- | --- | --- |
|  | **1996** | **2006** | **1996** | **2006** | **1996** | **2006** | **% Increase** |
| M 25-34 | 1,063,649 | 1,478,983 | 379 | 955 | 36 | 65 | 81.2 |
| M 35-44 | 652,453 | 1,053,893 | 542 | 1573 | 83 | 149 | 79.5 |
| M 45-54 | 381,580 | 636,889 | 522 | 1263 | 137 | 198 | 45.1 |
| M 55-64 | 269,255 | 351,702 | 743 | 1327 | 276 | 377 | 36.8 |
| M 65-74 | 171,321 | 213,778 | 1423 | 3000 | 830 | 1,404 | 69.0 |
| M 75-84 | 53,271 | 94,412 | 475 | 1471 | 893 | 1,558 | 74.6 |
| M 85+ | 8,803 | 13,020 | 107 | 203 | 1,221 | 1,558 | 27.6 |
| F 25-34 | 1,019,177 | 1,432,147 | 145 | 315 | 14 | 22 | 54.6 |
| F 35-44 | 634,984 | 1,016,763 | 195 | 517 | 31 | 51 | 65.5 |
| F 45-54 | 381,124 | 627,629 | 256 | 680 | 67 | 108 | 61.2 |
| F 55-64 | 271,637 | 363,840 | 314 | 604 | 116 | 166 | 43.5 |
| F 65-74 | 168,367 | 234,341 | 911 | 2268 | 541 | 968 | 78.9 |
| F 75-84 | 55,282 | 108,729 | 517 | 1764 | 936 | 1,622 | 73.4 |
| F 85+ | 11,427 | 18,365 | 125 | 298 | 1,094 | 1,622 | 48.2 |

**Part III- Risk Factors Profile**

***Table 3 – Risk Factors Profile***

|  | **Smoking Prevalence** | | | **Systolic Blood Pressure (mm Hg)** | | |
| --- | --- | --- | --- | --- | --- | --- |
|  | **SYRIA^*1^**  **1996** | **SYRIA^*2^**  **2006** | **Change** | **SYRIA^*1^**  **1996** | **SYRIA^*2^**  **2006** | **Change** |
| M 25-34 | 0.660 | 0.699 | 0.038 | 119.2 | 124.7 | 5.527 |
| M 35-44 | 0.586 | 0.620 | 0.034 | 120.8 | 129.0 | 8.173 |
| M 45-54 | 0.491 | 0.520 | 0.029 | 127.7 | 134.1 | 6.366 |
| M 55-64 | 0.358 | 0.378 | 0.021 | 138.1 | 143.3 | 5.270 |
| M 65-74 | 0.294 | 0.311 | 0.017 | 151.0 | 156.8 | 5.764 |
| M 75-84 | 0.294 | 0.311 | 0.017 | 151.0 | 156.8 | 5.764 |
| M 85+ | 0.294 | 0.311 | 0.017 | 151.0 | 156.8 | 5.764 |
| F 25-34 | 0.078 | 0.115 | 0.037 | 108.6 | 120.1 | 11.497 |
| F 35-44 | 0.164 | 0.241 | 0.077 | 112.1 | 127.4 | 15.313 |
| F 45-54 | 0.114 | 0.167 | 0.053 | 127.9 | 136.1 | 8.212 |
| F 55-64 | 0.065 | 0.096 | 0.031 | 137.9 | 144.1 | 6.183 |
| F 65-74 | 0.056 | 0.081 | 0.026 | 142.8 | 149.2 | 6.403 |
| F 75-84 | 0.012 | 0.018 | 0.006 | 142.8 | 154.2 | 11.438 |
| F 85+ | 0.012 | 0.018 | 0.006 | 142.8 | 154.2 | 11.438 |
|  |  |  |  |  |  |  |
|  | **Cholesterol (mmol/L)** | | | **BMI** | | |
|  | **SYRIA^*1^**  **1996** | **SYRIA^*2^**  **2006** | **Change** | **SYRIA^*1^**  **1996** | **SYRIA^*2^**  **2006** | **Change** |
| M 25-34 | 4.62 | 4.87 | 0.246 | 26.02 | 26.6 | 0.588 |
| M 35-44 | 5.18 | 5.31 | 0.128 | 26.52 | 28.6 | 2.088 |
| M 45-54 | 5.25 | 5.53 | 0.279 | 28.04 | 30.0 | 2.006 |
| M 55-64 | 5.32 | 5.44 | 0.122 | 27.63 | 29.5 | 1.830 |
| M 65-74 | 5.52 | 5.84 | 0.315 | 27.63 | 29.9 | 2.275 |
| M 75-84 | 5.52 | 5.84 | 0.315 | 27.63 | 29.9 | 2.275 |
| M 85+ | 5.52 | 5.84 | 0.315 | 27.63 | 29.9 | 2.275 |
| F 25-34 | 4.66 | 4.96 | 0.304 | 27.53 | 27.5 | -0.057 |
| F 35-44 | 4.97 | 5.43 | 0.453 | 28.86 | 31.2 | 2.302 |
| F 45-54 | 5.38 | 5.94 | 0.562 | 31.08 | 35.7 | 4.602 |
| F 55-64 | 5.79 | 6.07 | 0.281 | 30.97 | 35.9 | 4.967 |
| F 65-74 | 5.79 | 6.04 | 0.254 | 30.97 | 33.9 | 2.959 |
| F 75-84 | 5.79 | 6.04 | 0.254 | 30.97 | 33.9 | 2.959 |
| F 85+ | 5.79 | 6.04 | 0.254 | 30.97 | 33.9 | 2.959 |
|  |  |  |  |  |  |  |
|  | **Diabetes Prevalence** | | |  |  |  |
|  | **SYRIA^*1^**  **1996** | **SYRIA^*2^**  **2006** | **Change** |  |  |  |
| M 25-34 | 0.000 | 0.000 | 0.000 |  |  |  |
| M 35-44 | 0.059 | 0.049 | -0.011 |  |  |  |
| M 45-54 | 0.171 | 0.249 | 0.078 |  |  |  |
| M 55-64 | 0.157 | 0.307 | 0.150 |  |  |  |
| M 65-74 | 0.193 | 0.376 | 0.183 |  |  |  |
| M 75-84 | 0.205 | 0.400 | 0.195 |  |  |  |
| M 85+ | 0.205 | 0.400 | 0.195 |  |  |  |
| F 25-34 | 0.013 | 0.027 | 0.014 |  |  |  |
| F 35-44 | 0.053 | 0.066 | 0.013 |  |  |  |
| F 45-54 | 0.168 | 0.272 | 0.104 |  |  |  |
| F 55-64 | 0.289 | 0.428 | 0.139 |  |  |  |
| F 65-74 | 0.289 | 0.428 | 0.139 |  |  |  |
| F 75-84 | 0.289 | 0.428 | 0.139 |  |  |  |
| F 85+ | 0.289 | 0.428 | 0.139 |  |  |  |

*1: based on Palestine data

*2: Aleppo diabetes survey 2006, support by- Stepwise Survey 2003

**Part IV- PATIENT GROUPS & TREATMENTS**

**Table 4: Estimated number of patients**

**Hospital admissions**

|  | **Hospital AMI^*1^** | **Unstable Angina^*2^** | **Heart Failure^*3^** | **CABG for Chronic angina** | |
| --- | --- | --- | --- | --- | --- |
|  |  |  |  | Most recent  5 y | between 5  and 10 y |
| M 25-34 | 441 | 732 | 235 | 3,897 | 2,301 |
| M 35-44 | 1566 | 2018 | 319 | 6,089 | 3,596 |
| M 45-54 | 4457 | 4443 | 873 | 16,502 | 9,744 |
| M 55-64 | 3711 | 3193 | 1074 | 15,925 | 9,404 |
| M 65-74 | 2641 | 2021 | 778 | 13,019 | 7,688 |
| M 75-84 | 1398 | 965 | 543 | 3,051 | 1,801 |
| M 85+ | 153 | 105 | 59 | 421 | 248 |
| F 25-34 | 331 | 210 | 114 | 0 | 1 |
| F 35-44 | 113 | 861 | 152 | 11,819 | 6,979 |
| F 45-54 | 907 | 2369 | 404 | 10,831 | 6,396 |
| F 55-64 | 930 | 1957 | 1,037 | 10,776 | 6,363 |
| F 65-74 | 816 | 2301 | 827 | 11,434 | 6,752 |
| F 75-84 | 451 | 1428 | 429 | 2,354 | 1,390 |
| F 85+ | 86 | 272 | 82 | 398 | 235 |
|  | 18,002 | 22,877 | 6,927 | 106,517 | 62,898 |

^*1^: AMI - Emergency admissions

^*2^: Unstable Angina – Emergency admissions

*3: Heart failure admitted to hospitals

Community cases

|  | **Chronic Angina** | **Heart Failure** | **Secondary prevention post MI** | |
| --- | --- | --- | --- | --- |
|  |  |  | **Last 5 years** | **Previous 5 years** |
| M 25-34 | 13,073 | 59 | 1918 | 15 |
| M 35-44 | 20,427 | 139 | 6815 | 55 |
| M 45-54 | 35,048 | 2,408 | 19397 | 157 |
| M 55-64 | 30,108 | 3,999 | 16148 | 130 |
| M 65-74 | 24,614 | 7,672 | 11492 | 93 |
| M 75-84 | 13,658 | 7,073 | 6084 | 49 |
| M 85+ | 1,884 | 975 | 664 | 5 |
| F 25-34 | 32,589 | 102 | 1442 | 12 |
| F 35-44 | 40,085 | 256 | 493 | 4 |
| F 45-54 | 35,205 | 808 | 3949 | 32 |
| F 55-64 | 33,623 | 2,841 | 4049 | 33 |
| F 65-74 | 25,262 | 13,920 | 3553 | 29 |
| F 75-84 | 12,318 | 10,265 | 1963 | 16 |
| F 85+ | 2,081 | 1,734 | 374 | 3 |
|  | 319,974 | 52,251 | 78,343 | 633 |

**Table 5: Estimated number of patients receiving treatments for primary prevention**

|  | **Statins** | **Antihypertensive**  **medication** |
| --- | --- | --- |
| M 25-34 | 0 | 5,869 |
| M 35-44 | 29,480 | 17,939 |
| M 45-54 | 58,307 | 51,747 |
| M 55-64 | 50,243 | 66,991 |
| M 65-74 | 5,778 | 40,720 |
| M 75-84 | 2,552 | 17,983 |
| M 85+ | 352 | 2,480 |
| F 25-34 | 0 | 32,029 |
| F 35-44 | 28,885 | 67,286 |
| F 45-54 | 59,774 | 202,712 |
| F 55-64 | 45,480 | 179,428 |
| F 65-74 | 17,147 | 115,565 |
| F 75-84 | 7,956 | 53,620 |
| F 85+ | 1,344 | 9,057 |
|  | 307,297 | 863,425 |

**Table 6: treatment uptake levels estimation in different CHD cases**

**Acute myocardial infarction**

|  | **Aspirin** | **ACE**  **inhibitors** | **Beta-blockers** | **PTCA (STEMI)** | **PTCA (NSTEMI)** | **CPR in hospital** | **Thrombolysis** |
| --- | --- | --- | --- | --- | --- | --- | --- |
| M 25-34 | 1.000 | 0.611 | 0.714 | 0.000 | 0.000 | 0.050 | 0.714 |
| M 35-44 | 0.944 | 0.611 | 0.944 | 0.033 | 0.022 | 0.050 | 0.778 |
| M 45-54 | 1.000 | 0.739 | 0.913 | 0.013 | 0.009 | 0.050 | 0.652 |
| M 55-64 | 0.895 | 0.737 | 0.895 | 0.000 | 0.000 | 0.050 | 0.553 |
| M 65-74 | 1.000 | 0.846 | 0.846 | 0.000 | 0.000 | 0.050 | 0.485 |
| M 75-84 | 1.000 | 0.846 | 0.417 | 0.000 | 0.000 | 0.050 | 0.417 |
| M 85+ | 1.000 | 0.846 | 0.417 | 0.000 | 0.000 | 0.050 | 0.417 |
| F 25-34 | 0.250 | 0.500 | 0.500 | 0.000 | 0.000 | 0.050 | 0.250 |
| F 35-44 | 0.500 | 0.500 | 0.661 | 0.000 | 0.000 | 0.050 | 0.475 |
| F 45-54 | 1.000 | 0.800 | 0.900 | 0.000 | 0.000 | 0.050 | 0.700 |
| F 55-64 | 1.000 | 0.789 | 0.792 | 0.000 | 0.000 | 0.050 | 0.667 |
| F 65-74 | 1.000 | 0.778 | 0.792 | 0.000 | 0.000 | 0.050 | 0.556 |
| F 75-84 | 1.000 | 0.500 | 0.390 | 0.000 | 0.000 | 0.050 | 0.556 |
| F 85+ | 1.000 | 0.500 | 0.390 | 0.000 | 0.000 | 0.050 | 0.556 |

**Unstable angina**

|  | **Aspirin** | **Aspirin & Heparin** | **PG IIA/IIB** | **CABG** | **PTCA** |
| --- | --- | --- | --- | --- | --- |
| M 25-34 | 0.125 | 0.875 | 0.000 | 0.000 | 0.000 |
| M 35-44 | 0.091 | 0.909 | 0.000 | 0.000 | 0.000 |
| M 45-54 | 0.040 | 0.960 | 0.000 | 0.000 | 0.000 |
| M 55-64 | 0.025 | 0.975 | 0.000 | 0.000 | 0.000 |
| M 65-74 | 0.013 | 0.988 | 0.000 | 0.000 | 0.000 |
| M 75-84 | 0.000 | 1.000 | 0.000 | 0.000 | 0.000 |
| M 85+ | 0.000 | 1.000 | 0.000 | 0.000 | 0.000 |
| F 25-34 | 0.091 | 0.909 | 0.000 | 0.000 | 0.000 |
| F 35-44 | 0.091 | 0.909 | 0.000 | 0.000 | 0.000 |
| F 45-54 | 0.037 | 0.963 | 0.000 | 0.000 | 0.000 |
| F 55-64 | 0.054 | 0.946 | 0.000 | 0.000 | 0.000 |
| F 65-74 | 0.054 | 0.946 | 0.000 | 0.000 | 0.000 |
| F 75-84 | 0.000 | 1.000 | 0.000 | 0.000 | 0.000 |
| F 85+ | 0.000 | 1.000 | 0.000 | 0.000 | 0.000 |

**2' prevention following MI - 2000-2006**

|  | **Statins** | **Aspirin** | **Warfarin** | **ACE inhibitors** | **Beta-blockers** |
| --- | --- | --- | --- | --- | --- |
| M 25-34 | 0.143 | 0.333 | 0.04 | 0.21 | 0.61 |
| M 35-44 | 0.306 | 0.409 | 0.04 | 0.36 | 0.61 |
| M 45-54 | 0.370 | 0.519 | 0.04 | 0.38 | 0.63 |
| M 55-64 | 0.289 | 0.438 | 0.04 | 0.38 | 0.55 |
| M 65-74 | 0.288 | 0.390 | 0.04 | 0.28 | 0.31 |
| M 75-84 | 0.208 | 0.357 | 0.04 | 0.18 | 0.08 |
| M 85+ | 0.208 | 0.357 | 0.04 | 0.18 | 0.08 |
| F 25-34 | 0.125 | 0.125 | 0.06 | 0.18 | 0.50 |
| F 35-44 | 0.138 | 0.287 | 0.06 | 0.24 | 0.50 |
| F 45-54 | 0.150 | 0.449 | 0.06 | 0.29 | 0.50 |
| F 55-64 | 0.292 | 0.634 | 0.06 | 0.30 | 0.38 |
| F 65-74 | 0.222 | 0.692 | 0.06 | 0.40 | 0.35 |
| F 75-84 | 0.167 | 0.750 | 0.06 | 0.24 | 0.33 |
| F 85+ | 0.167 | 0.750 | 0.06 | 0.24 | 0.33 |

**2' prevention following MI - 1995-1999**

|  | **Statins** | **Aspirin** | **Warfarin** | **ACE inhibitors** | **Beta-blockers** |
| --- | --- | --- | --- | --- | --- |
| M 25-34 | 0.00 | 0.333 | 0.04 | 0.05 | 0.61 |
| M 35-44 | 0.00 | 0.409 | 0.04 | 0.09 | 0.61 |
| M 45-54 | 0.00 | 0.519 | 0.04 | 0.09 | 0.63 |
| M 55-64 | 0.00 | 0.438 | 0.04 | 0.10 | 0.55 |
| M 65-74 | 0.00 | 0.390 | 0.04 | 0.07 | 0.31 |
| M 75-84 | 0.00 | 0.357 | 0.04 | 0.05 | 0.08 |
| M 85+ | 0.00 | 0.357 | 0.04 | 0.05 | 0.08 |
| F 25-34 | 0.00 | 0.125 | 0.06 | 0.05 | 0.50 |
| F 35-44 | 0.00 | 0.287 | 0.06 | 0.06 | 0.50 |
| F 45-54 | 0.00 | 0.449 | 0.06 | 0.07 | 0.50 |
| F 55-64 | 0.00 | 0.634 | 0.06 | 0.08 | 0.38 |
| F 65-74 | 0.00 | 0.692 | 0.06 | 0.10 | 0.35 |
| F 75-84 | 0.00 | 0.750 | 0.06 | 0.06 | 0.33 |
| F 85+ | 0.00 | 0.750 | 0.06 | 0.06 | 0.33 |

**2' prevention following CABG / PTCA**

|  | **Statins** | **Aspirin** | **Warfarin** | **ACE inhibitors** | **Beta-blockers** |
| --- | --- | --- | --- | --- | --- |
| M 25-34 | 0.62 | 1.00 | 0.01 | 0.16 | 1.00 |
| M 35-44 | 0.62 | 1.00 | 0.01 | 0.16 | 1.00 |
| M 45-54 | 0.62 | 0.88 | 0.01 | 0.16 | 0.94 |
| M 55-64 | 0.88 | 0.75 | 0.01 | 0.16 | 0.88 |
| M 65-74 | 0.50 | 0.50 | 0.01 | 0.16 | 0.38 |
| M 75-84 | 0.43 | 0.57 | 0.01 | 0.16 | 0.43 |
| M 85+ | 0.43 | 0.57 | 0.01 | 0.16 | 0.43 |
| F 25-34 | 0.50 | 1.00 | 0.01 | 0.16 | 1.00 |
| F 35-44 | 0.50 | 1.00 | 0.01 | 0.16 | 1.00 |
| F 45-54 | 0.50 | 0.88 | 0.01 | 0.16 | 1.00 |
| F 55-64 | 0.50 | 0.75 | 0.01 | 0.16 | 1.00 |
| F 65-74 | 0.50 | 0.50 | 0.01 | 0.16 | 0.50 |
| F 75-84 | 0.50 | 0.57 | 0.01 | 0.16 | 0.50 |
| F 85+ | 0.50 | 0.57 | 0.01 | 0.16 | 0.50 |

**Chronic angina**

|  | **Statins** | **Aspirin** | **CABG** | **PTCA** |
| --- | --- | --- | --- | --- |
| M 25-34 | 0.212 | 0.333 | 0.081 | 0.071 |
| M 35-44 | 0.295 | 0.409 | 0.081 | 0.074 |
| M 45-54 | 0.316 | 0.519 | 0.128 | 0.097 |
| M 55-64 | 0.336 | 0.438 | 0.144 | 0.106 |
| M 65-74 | 0.347 | 0.390 | 0.144 | 0.104 |
| M 75-84 | 0.357 | 0.357 | 0.061 | 0.091 |
| M 85+ | 0.357 | 0.357 | 0.061 | 0.091 |
| F 25-34 | 0.125 | 0.125 | 0.000 | 0.000 |
| F 35-44 | 0.286 | 0.287 | 0.080 | 0.080 |
| F 45-54 | 0.306 | 0.449 | 0.083 | 0.084 |
| F 55-64 | 0.465 | 0.634 | 0.087 | 0.070 |
| F 65-74 | 0.524 | 0.692 | 0.123 | 0.070 |
| F 75-84 | 0.583 | 0.750 | 0.052 | 0.000 |
| F 85+ | 0.583 | 0.750 | 0.052 | 0.000 |

**Heart failure with hospital admission**

|  | **Statins** | **Aspirin** | **ACE inhibitors** | **Beta-blockers** | **Spironolactone** |
| --- | --- | --- | --- | --- | --- |
| M 25-34 | 0.167 | 0.667 | 0.050 | 0.267 | 0.333 |
| M 35-44 | 0.500 | 0.750 | 0.050 | 0.267 | 0.458 |
| M 45-54 | 0.500 | 0.833 | 0.750 | 0.267 | 0.583 |
| M 55-64 | 0.571 | 0.786 | 0.643 | 0.357 | 0.786 |
| M 65-74 | 0.615 | 0.769 | 0.462 | 0.077 | 0.462 |
| M 75-84 | 0.615 | 0.833 | 0.750 | 0.083 | 0.417 |
| M 85+ | 0.615 | 0.833 | 0.750 | 0.083 | 0.417 |
| F 25-34 | 0.667 | 0.778 | 0.278 | 0.143 | 0.222 |
| F 35-44 | 0.667 | 0.778 | 0.278 | 0.143 | 0.222 |
| F 45-54 | 0.667 | 0.778 | 0.556 | 0.333 | 0.444 |
| F 55-64 | 0.643 | 0.786 | 0.500 | 0.214 | 0.429 |
| F 65-74 | 0.500 | 0.875 | 0.750 | 0.250 | 0.375 |
| F 75-84 | 0.429 | 0.857 | 0.571 | 0.143 | 0.429 |
| F 85+ | 0.429 | 0.857 | 0.571 | 0.143 | 0.429 |

**Heart failure in the community**

|  | **Statins** | **Aspirin** | **ACE inhibitors** | **Beta-blockers** | **Spironolactone** |
| --- | --- | --- | --- | --- | --- |
| M 25-34 | 0.3333 | 0.8889 | 0.870 | 0.667 | 0.81159 |
| M 35-44 | 0.5000 | 0.8889 | 0.870 | 0.667 | 0.81159 |
| M 45-54 | 0.6667 | 0.8889 | 0.870 | 0.556 | 0.81159 |
| M 55-64 | 0.6471 | 0.8235 | 0.870 | 0.471 | 0.81159 |
| M 65-74 | 0.7895 | 0.6250 | 0.870 | 0.458 | 0.81159 |
| M 75-84 | 0.6154 | 0.6250 | 0.870 | 0.231 | 0.81159 |
| M 85+ | 0.6154 | 0.6250 | 0.870 | 0.231 | 0.81159 |
| F 25-34 | 0.4444 | 0.6667 | 0.500 | 0.500 | 0.64815 |
| F 35-44 | 0.4444 | 0.6667 | 0.694 | 0.500 | 0.64815 |
| F 45-54 | 0.4444 | 0.6667 | 0.889 | 0.444 | 0.64815 |
| F 55-64 | 0.6667 | 0.6667 | 0.833 | 0.333 | 0.64815 |
| F 65-74 | 0.5833 | 0.6333 | 0.633 | 0.259 | 0.64815 |
| F 75-84 | 0.5000 | 0.6000 | 0.600 | 0.259 | 0.64815 |
| F 85+ | 0.5000 | 0.6000 | 0.600 | 0.259 | 0.64815 |

**Primary prevention**

|  | **Statins for primary prevention** | **Antihypertensive medication** |
| --- | --- | --- |
| M 25-34 | 0.000 | 0.011 |
| M 35-44 | 0.028 | 0.045 |
| M 45-54 | 0.092 | 0.148 |
| M 55-64 | 0.143 | 0.281 |
| M 65-74 | 0.027 | 0.281 |
| M 75-84 | 0.027 | 0.281 |
| M 85+ | 0.027 | 0.281 |
| F 25-34 | 0.000 | 0.029 |
| F 35-44 | 0.028 | 0.106 |
| F 45-54 | 0.095 | 0.240 |
| F 55-64 | 0.125 | 0.374 |
| F 65-74 | 0.073 | 0.374 |
| F 75-84 | 0.073 | 0.374 |
| F 85+ | 0.073 | 0.374 |

# Table 7: Clinical efficacy of interventions: relative risk reductions obtained from meta-analyses, and randomised clinical trials

| **Treatments** | **Relative risk reduction^†^** | **Comments** | **Source paper: First author (year), notes** |
| --- | --- | --- | --- |
| ***ST elevation myocardial infarction (STEMI)*** | | | |
|  |  |  |  |
| **Thrombolysis** | 31% (95% CI: 14,45) | <55 years: Odds Ratio (OR)=0.692; Relative Risk Reduction (RRR)=30.8% (95% CI: 14,45)  55-64 years: OR=0.736; RRR=26.4% (95% CI: 17,40)  65-74 years: OR=0.752; RRR=24.8% (95% CI: 15,37)  > 75 years: OR=0.844; RRR=15.6% (95% CI: 4,30) | Estess (2002)^25^ |
| **Aspirin** | 23% (95% CI: 15,30) | RRR=23% (95% CI: 15,30): outcome is vascular deaths | ISIS-2 (1988)^26^ |
| **Primary CABG surgery** | 39% (95% CI: 23,52) | OR=0.61 (95% CI: 0.48,0.77); RRR=39% (95% CI: 23,52) on page 565, 0-5 year mortality | Yusuf (1994)^27^ |
| **Primary PCI** | 30% (95% CI: 15,42) | OR=0.70 (95% CI: 0.58,0.85); RRR=30% (95% CI: 15,42) outcome compares primary angioplasty to thrombolytics. | Keeley (2003)^28^ |
| **Beta blockers** | 4% (95% CI: -8,15) | OR=0.96 (95% CI: 0.85,1.08); RRR=4% (95% CI: -8,15) on page 1732 | Freemantle (1999)^29^ |
| **ACE inhibitors** | 7% (95% CI: 2,11) | OR=0.93 (95% CI: 0.89,0.98); RRR=7% (95% CI: 2,11) for 30 day mortality in myocardial infarction | ACE Inhibitor Myocardial Infarction Collaborative Group (1998)^30^ |
| **Clopidogrel** | 3% (95% CI: 1,6) | RRR=3% (95% CI: 1,6) for 30 day mortality in myocardial infarction | Chen (2005)^31^  Sabatine (2005)^32^ |
| **Hospital CPR** | 33% (95% CI: 10,36) | Survival at 24 hours estimated to be 32%, discharge to home at 21%, and 1 year survival to be 15% overall. | Tunstall-Pedoe (1992)^33^  Nadkarni ^34^ |
| ***Non-ST-segment elevation acute coronary syndrome (NSTEACS):*** | | | |
|  |  |  |  |
| **Aspirin alone** | 15% (95% CI: 11,19) | OR=0.85 (95% CI: 0.49,0.95); RRR=15% (95% CI: 11,19). Outcome is vascular and nonvascular deaths on page 75. Assume appropriate for patients with NSTE-ACS. | Antithrombotic Trialists’ Collaboration (2002)^35^ |
| **Aspirin & heparin** | 33% (95% CI: -2,56) | OR=0.67 (95% CI: 0.48,1.02); RRR=33% (95% CI: -2,56%) in Table 2. The study outcome is composite MI death and non-fatal MI; compares those on aspirin & heparin to aspirin only. | Oler (1996)^36^ |
| **Platelet glycoprotein IIB/IIIA inhibitors** | 9% (95% CI: 2,16) | OR=0.91 (95% CI: 0.84,0.98); RRR=9% (95% CI: 2,16). Study looked at acute coronary syndrome without persistent ST elevation. | Boersma (2002)^37^ |
| **Early PCI** | 32% (95% CI: 5,51) | OR=0.68 (95% CI: 0.49,0.95); RRR=32% (95% CI: 5,51) | RITA 3 (Fox 2005)^38^ |
| **Primary CABG surgery** | 39% (95% CI: 23,52) | OR=0.61 (95% CI: 0.48,0.77); RRR=39% (95% CI: 23,52) on page 565, 0-5 year mortality | Yusuf (1994)^27^.  Assumed similar as STEMI. |
| **Clopidogrel** | 7% (95% CI: 2,11) | RRR=7% (95% CI: 2,11) | Yusuf (2001)^39^ |
| **Beta blockers** | 4% (95% CI: -8,15) | OR=0.96 (95% CI: 0.85,1.08); RRR=4% (95% CI: -8,15) on page 1732 | Freemantle (1999)^29^  Assumed similar as STEMI. |
| **ACE inhibitors** | 7% (95% CI: 2,11) | OR=0.93 (95% CI: 0.89,0.98); RRR=7% (95% CI: 2,11) for 30 day mortality in myocardial infarction | ACE Inhibitor Myocardial Infarction Collaborative Group (1998)^30^ |
| ***Secondary prevention post myocardial infarction/revascularisation:*** | | | |
|  |  |  |  |
| **Aspirin** | 15% (95% CI: 11,19) | OR=0.85 (95% CI: 0.49,0.95); RRR=15% (95% CI: 11,19). Outcome is vascular and nonvascular deaths on page 75. This data seems to be appropriate to this outcome in CHD patients. | Antithrombotic Trialists’ Collaboration (2002)^35^ |
| **Beta blockers** | 23% (95% CI: 15,31) | OR=0.77 (95% CI: 0.69,0.85); RRR=23% (95% CI: 15,31) on page 1734. Odds of death in long term trials. | Freemantle (1999)^29^ |
| **ACE inhibitors or Angiotensin-II receptor antagonists** | 20% (95% CI: 13,26) | OR=0.80 (95% CI: 0.74,0.87); RRR=20% (95% CI: 13,26) on page 1577, death up to four years [endpoint of study looking at those with heart failure or LV dysfunction]. | Flather (2000)^40^ |
| **Statins** | 24% (95% CI: 10,26) | RRR=24% (95% CI: 10,26)  Intensive statin therapy in acute coronary syndromes. | Hulten (2006)^41^ |
| **Warfarin** | 22% (95% CI: 13,31) | OR=0.78 (95% CI: 0.67,0.90); RRR=22% (95% CI: 10,33) | Anand and Yusuf (1999)^42^ |
| **Rehabilitation** | 26% (95% CI: 10,39) | OR=0.74 (95% CI: 0.61,0.90); RRR=26% (95% CI: 10,39) in Figure 1, page 685 Taylor reference | Taylor (2004)^43^ |
| ***Chronic stable coronary artery disease:*** | | | |
|  |  |  |  |
| **CABG surgery**  **years 0-5** | 39% (95% CI:23,52) | OR = 0.61 (95% CI: 0.48-0.77), RRR 39% (95% CI: 23,52) on page 565, 5 year mortality | Yusuf (1994)^27^ |
| **CABG surgery**  **years 6-10** | 32% (95% CI: 2,30) | OR = 0.83 (95% CI: 0.70-0.98), RRR 17% (95% CI: 2,30) on page 565, 10 year mortality.  OR = 0.68 (95% CI: 0.56-0.83), RRR 32% (95% CI: 17,44) on page 565, 7 year mortality  CABG compared to medical treatment | Yusuf (1994)^27^ |
| **Angioplasty** | No effect |  | Boden (2007) ^44^ |
| **Aspirin** | 15% (95% CI: 11,19) | OR=0.85 (95% CI: 0.49-0.95); RRR=15% (95% CI: 11,19). Outcome is vascular and nonvascular deaths on page 75. | Antithrombotic Trialists’ Collaboration (2002)^35^ |
| **Statins** | 23% (95% CI: 10,26) | RRR=23% (95% CI 10,26)  Standard dose statin therapy in coronary artery disease. | Wilt (2004)^45^ |
| **ACE inhibitors/ARB** | 17% (95% CI: 6,28) | RRR=17% (95% CI 6,28) | Al-Mallah (2006)^46^ |
| ***Heart failure in patients requiring hospitalisation or in the community:*** | | | |
|  |  |  |  |
| **ACE inhibitors** | 20% (95% CI: 13,26) | OR=0.80 (95% CI: 0.74,0.87); RRR=20% (95% CI: 13,26) on page 1577 [death up to four years was study endpoint for those with heart failure or LV dysfunction] | Flather (2000)^40^ |
| **Beta blockers** | 35% (95% CI: 26,43) | OR=0.65 (95% CI: 0.57,0.74); RRR=35% (95% CI: 26,43): all cause mortality | Shibata (2001)^47^ |
| **Spironolactone** | 30% (95% CI: 18,41)  31% (95% CI: 18,42) | OR=0.70 (95% CI: 0.59,0.82); RRR=30% (95% CI: 18,41) in those that had at least one cardiac related hospitalisation.  OR=0.69 (95% CI: 0.58,0.82); RRR=31% (95% CI: 18,42) in entire study population consisting of those with community heart failure, page 711. | Pitt (1999)^48^ |
| **Aspirin** | 15% (95% CI: 11,19) | OR=0.85 (95% CI: 0.49,0.95); RRR=15% (95% CI: 11,19). Outcome is vascular and nonvascular deaths on page 75. | Antithrombotic Trialists’ Collaboration (2002)^35^ |
| **Statins** | No effect |  | Kjekshus (2007)^49^  Tavazzi (2008)^50^ |
| ***Primary prevention therapies:*** | | | |
|  |  |  |  |
| **Treatments for high blood pressure** | 13% (95% CI: 6,19) | OR=0.87 (95% CI: 0.81,0.94); RRR=13% (95% CI: 6,19) in those with high blood pressure without disease at entry. [RRR=29% (95% CI: 17,37) those with average blood pressure and CHD, treated with ACE inhibitors] | Law (2003)^51^ |
| **Statins** | 35% (95% CI: 11,52) | OR=0.65 (95% CI: 0.48,0.89); RRR=35% (95% CI: 11,52) for CHD mortality (only trials using statins), Figure 3 on page 4 | Pignone (2000)^52^ |

^†^Relative risk reduction (RRR) calculated as 1 – odds ratio

# Reference List

(1) Unal B, Critchley JA, Capewell S. Explaining the decline in coronary heart disease mortality in England and Wales between 1981 and 2000. Circulation 2004;109:1101-7.

(2) Ford ES, Ajani UA, Croft JB, Critchley JA, Labarthe DR, Kottke TE et al. Explaining the decrease in US deaths from coronary disease, 1980-2000. New England Journal of Medicine 2007;356:2388-98. Supplementary Appendix available at http://www.nejm.org/doi/suppl/10.1056/NEJMsa053935/suppl_file/nejm_ford_2388sa1.pdf

(3) Capewell S, Beaglehole R, Seddon M, McMurray J. Explanation for the decline in coronary heart disease mortality rates in Auckland, New Zealand, between 1982 and 1993. Circulation 2000;102:1511-6.

(4) Capewell S, Morrison CE, McMurray JJ. Contribution of modern cardiovascular treatment and risk factor changes to the decline in coronary heart disease mortality in Scotland between 1975 and 1994. Heart 1999;81:380-6.

(5) Wijeysundera H.C, Machado M, Farahati F, Wang X, Witteman W, van der Valde G et al. Association of temporal trends in risk factors and treatment uptake with coronary heart disease mortality, 1994-2005. Journal of the American Medical Association 2010;303:1841-7.

(6) Yeh R.W., Sidney S, Chandra M, Sorel M, Selby J.V., Go A.S. Population trends in the incidence and outcomes of acute myocardial infarction. The New England Journal of Medicine 2010;362:2155-65.

(7) Dauchet L, Amouyel P, Hercberg S, Dallongeville J. Fruit and vegetable consumption and risk of coronary heart disease: A meta-analysis of cohort studies. Journal of Nutrition 2006;136:2588-93.

(8) Porta M. A dictionary of epidemiology. Oxford University Press; 2008.

(9) Ezatti M, Lopez AD, Rodgers A, Murray CJL, editors. Comparative quantification of risk. Global and regional burden of disease attributable to selected major risk factors. World Health Organization; 2004.

(10) Roglic G, Unwin N. Mortality attributable to diabetes: estimates for the year 2010. Diabetes research and clinical practice 2010;87:15-19.

(11) Huxley R, Barzi F, Woodward M. Excess risk of fatal coronary heart disease associated with diabetes in men and women: meta-analysis of 37 prospective cohort studies. BMJ 2006;332(7533):73-78.

(12) Danaei G, Ding EL, Mozaffarian D, Taylor B, Rehm J, et al (2009) The Preventable Causes of Death in the United States: Comparative Risk Assessment of Dietary, Lifestyle, and Metabolic Risk Factors. PLoS Med 6(4): e1000058.

(13) Tobias M, Taylor R, Yeh LC, Huang K, Mann S, Sharpe N. Did it fall or was it pushed? The contribution of trends in established risk factors to the decline in premature coronary heart disease mortality in New Zealand. Aust N Z J Public Health 2008;32:117-25.

(14) Taylor R, Dobson A, Mirzaei M. Contribution of changes in risk factors to the decline of coronary heart disease mortality in Australia over three decades. European Journal of Cardiovascular Prevention and Rehabilitation 2006;13:760-768.

(15) Dobson A, McElduff P, Heller R, Alexander H, Colley P, D'Este K. Changing patterns of coronary heart disease in the Hunter region of New South Wales, Australia. J Clin Epidemiol 1999;52:761-771.

(16) Yusuf S. Two decades of progress in preventing vascular disease. Lancet 2002;360:2-3.

(17) Wald NJ, Law MR. A strategy to reduce cardiovascular disease by more than 80%. British Medical Journal 2003;326:1419-1424.

(18) Mant J, Hicks N. Detecting Differences in Quality of Care - the Sensitivity of Measures of Process and Outcome in Treating Acute Myocardial-Infarction. British Medical Journal 1995;311:793-6.

(19) Noble M, mcLennan D, Wilkinson K et al. The English Indices of Deprivation 2007. Department for Communities and Local Government.

(20) Adams J, White M. Removing the health domain from the Index of Multiple Deprivation 2004 - effect of measured inequalities in census measure of health. Journal of Public Health 2006;28:379-83.

(21) DH Vascular Programme Team. Treatment of heart Attack National Guidance. Final Report of the National Infarct Angioplasty Project (NIAP). 2008.

(22) National Audit Team British Heart Foundation, University of York. The National Audit of Cardiac Rehabilitation. Annual Statistical report 2008. London: British Heart Foundation; 2007.

(23) Nicol ED, Fittall B, Roughton M, Cleland JGF, Dargie H, Cowie MR. NHS heart failure survey: a survey of acute heart failure admissions in England, Wales and Northern Ireland. Heart 2008;94:172-7.

(24) Craig R, Mindell J. Health Survey for England 2006. 2008. London, United Kingdom, The Information Centre.

(25) Estess JM, Topol EJ. Fibrinolytic treatment for elderly patients with acute myocardial infarction. Heart 2002 April;87:308-11.

(26) ISIS-2 (Second international study of infarct survival) collaborative group. Randomised trial of intravenous streptokinase, oral aspirin, both, or neither among 17 187 cases of suspected acute myocardial infarction: ISIS-2. Lancet 1988;8607:349-60.

(27) Yusuf S, Zucker D, Peduzzi P, Fisher LD, Takaro T, Kennedy JW et al. Effect of Coronary-Artery Bypass Graft-Surgery on Survival - Overview of 10-Year Results from Randomized Trials by the Coronary-Artery Bypass Graft-Surgery Trialists Collaboration. Lancet 1994;344:563-70.

(28) Keeley EC, Boura JA, Grines CL. Primary angioplasty versus intravenous thrombolytic therapy for acute myocardial infarction: a quantitative review of 23 randomised trials. Lancet 2003;361:13-20.

(29) Freemantle N, Cleland J, Young P, Mason J, Harrison J. beta Blockade after myocardial infarction: systematic review and meta regression analysis. BMJ 1999 June 26;318:1730-7.

(30) Ace Inhibitor Myocardial Infarction Collaborative Group. Indications for ACE inhibitors in the early treatment of acute myocardial infarction: Systematic overview of individual data from 100 000 patients in randomized trials. Circulation 1998;97:2202-12.

(31) Chen ZM, Jiang LX, Chen YP, et al. Addition of clopidogrel to aspirin in 45,852 patients with acute myocardial infarction: randomised placebo-controlled trial. Lancet 2005;366:1607-21.

(32) Sabatine MS, Cannon CP, Gibson CM, Lopez-Sendon JL, Montalescot G, Theroux P et al. Addition of clopidogrel to aspirin and fibrinolytic therapy for myocardial infarction with ST-segment elevation. New England Journal of Medicine 2005;352:1179-89.

(33) Tunstall-Pedoe H, Bailey L, Chamberlain DA, Marsden AK, Ward ME, Zideman DA. Survey of 3765 cardiopulmonary resuscitations in British hospitals (the BRESUS Study): methods and overall results. BMJ 1992 May 23;304:1347-51.

(34) Nadkarni VM, Larkin GL, Peberdy MA, Carey SM, Kaye W, Mancini ME et al. First documented rhythm and clinical outcome from in-hospital cardiac arrest among children and adults. JAMA 2006 January 4;295:50-7.

(35) Antithrombotic Trialists' Collaboration. Collaborative meta-analysis of randomised trials of antiplatelet therapy for prevention of death, myocardial infarction, and stroke in high risk patients (vol 324, pg 71, 2002). British Medical Journal 2002;324:141.

(36) Oler A, Whooley MA, Oler J, Grady D. Adding heparin to aspirin reduces the incidence of myocardial infarction and death in patients with unstable angina. A meta-analysis. JAMA 1996 September 11;276:811-5.

(37) Boersma E, Harrington RA, Moliterno DJ, White H, Theroux P, Van de Werf F et al. Platelet glycoprotein IIb/IIIa inhibitors in acute coronary syndromes: a meta-analysis of all major randomised clinical trials. Lancet 2002;359:189-98.

(38) Fox KAA, Poole-Wilson P, Clayton TC, Henderson RA, Shaw TRD, Wheatley DJ et al. 5-year outcome of an interventional strategy in non-ST-elevation acute coronary syndrome: the British Heart Foundation RITA 3 randomised trial. Lancet 2005;366:914-20.

(39) Yusuf S, Zhao F, Mehta SR, Chrolavicius S, Tognoni G, Fox KK. Effects of clopidogrel in addition to aspirin in patients with acute coronary syndromes without ST-segment elevation. New England Journal of Medicine 2001;345:494-502.

(40) Flather MD, Yusuf S, Kober L, Pfeffer M, Hall A, Murray G et al. Long-term ACE-inhibitor therapy in patients with heart failure or left-ventricular dysfunction: a systematic overview of data from individual patients. ACE-Inhibitor Myocardial Infarction Collaborative Group. Lancet 2000 May 6;355:1575-81.

(41) Hulten E, Jackson JL, Douglas K, George S, Villines TC. The effect of early, intensive statin therapy on acute coronary syndrome: a meta-analysis of randomized controlled trials. Arch Intern Med 2006;166:1814-21.

(42) Anand SS, Yusuf S. Oral anticoagulant therapy in patients with coronary artery disease: a meta-analysis. JAMA 1999 December 1;282:2058-67.

(43) Taylor RS, Brown A, Ebrahim S, Jolliffe J, Noorani H, Rees K et al. Exercise-based rehabilitation for patients with coronary heart disease: systematic review and meta-analysis of randomized controlled trials. Am J Med 2004 May 15;116:682-92.

(44) Boden WE, O'Rourke RA, Teo KK, Hartigan PM, Maron DJ, Kostuk WJ et al. Optimal medical therapy with or without PCI for stable coronary disease. New England Journal of Medicine 2007;356:1503-16.

(45) Wilt TJ, Bloomfield HE, MacDonald R, et al. Effectiveness of statin therapy in adults with coronary heart disease. Arch Intern Med 2004;164:1427-36.

(46) Al-Mallah MH, Tleyjeh IM, Abdel-Latif AA, Weaver WD. Angiotensin-converting enzyme inhibitors in coronary artery disease and preserved left ventricular systolic function: a systematic review and meta-analysis of randomized controlled trials. Journal of the American College of Cardiology 2006;47:1576-83.

(47) Shibata MC, Flather MD, Wang DL. Systematic review of the impact of beta blockers on mortality and hospital admissions in heart failure. European Journal of Heart Failure 2001;3:351-7.

(48) Pitt B, Zannad F, Remme WJ, Cody R, Castaigne A, Perez A et al. The effect of spironolactone on morbidity and mortality in patients with severe heart failure. New England Journal of Medicine 1999;341:709-17.

(49) Kjekshus J, Apetrei E, Barrios V, et al. Rosuvastatin in older patients with systolic heart failure. New England Journal of Medicine 2007;357:2248-61.

(50) Tavazzi L, Maggioni AP, Marchioli R, et al. Effect of rosuvastatin in older patients with chronic heart failure (the GISSI-HF trial): a randomised, double-blind, placebo-controlled trial. Lancet 2008;372:1231-9.

(51) Law M, Wald N, Morris J. Lowering blood pressure to prevent myocardial infarction and stroke: a new preventive strategy. Health Technol Assess 2003;7:1-94.

(52) Pignone M, Phillips C, Mulrow C. Use of lipid lowering drugs for primary prevention of coronary heart disease: meta-analysis of randomised trials. British Medical Journal 2000;321:983-6.

(53) Lewington S, Clarke R, Qizilbash N, Peto R, Collins R. Age-specific relevance of usual blood pressure to vascular mortality: a meta-analysis of individual data for one million adults in 61 prospective studies. Lancet 2002 December 14;360:1903-13.

(54) Lewington S, Whitlock G, Clarke R, Sherliker P, Emberson J, Halsey J et al. Blood cholesterol and vascular mortality by age, sex, and blood pressure: a meta-analysis of individual data from 61 prospective studies with 55000 vascular deaths. Lancet 2007;370:1829-39.

(55) Bogers RP, Hoogenveen RT, Boshuizen H, Woodward M, Knekt P, Van Dam RM et al. Overweight and obesity increase the risk of coronary heart disease: A pooled analysis of 30 prospective studies. European Journal of Epidemiology 2006;21:313.

(56) James WPT, Jackson-Leach R, Mhurchu CN et al. Overweight and obesity (high body mass index). In: Ezatti M, Lopez AD, Rodgers A, Murray CJL, editors. Comparative quantification of risk. Global and regional burden of disease attributable to selected major risk factors. Volume 1 ed. World Health Organization; 2004. p. 497-596.

(57) Ezzati M, Henley SJ, Thun MJ, Lopez AD. Role of smoking in global and regional cardiovascular mortality. Circulation. 2005 112:489-497.

(58) Bull F, Armstrong TP, Dixon T, Ham S, Neiman A, Pratt M. Physical inactivity. In: Ezatti M, Lopez AD, Rodgers A, Murray CJL, editors. Comparative quantification of risk. Global and regional burden of disease attributable to selected major risk factors. Volume 1 ed. World Health Organization; 2004. p. 729-881.

(59) Joubert J, Norman R, Lambert EV, Groenewald P et al. Estimating the burden of disease attributable to physical inactivity in South Africa in 2000. South African Medical Journal 2007;97:725-731.

(60) Hu G, Qiao Q, Tuomilehto J, Balkau B, Borch-Johnsen K, Pyorala K, for the DECODE Study Group. Prevalence of the metabolic syndrome amd its relation to all-cause and cardiovascular mortality in nondiabetic European men and women. Arch Intern Med 164:2004;1066-1076.

(61) Morrish NJ, Wang SL, Stevens LK, Fuller JH, Keen H. Mortality and causes of death in the WHO Multinational Study of Vascular Disease in Diabetes. Diabetologia 2001 Sep;44 Suppl 2: S14-21.

(62) Barendregt JJ. The effect size in uncertainty analysis. Value in Health 2010;4:388-391.

(63) Weir R.A.P, McMurray J.J.V, Velazquez E.J. Epidemiology of heart failure and left ventricular systolic dysfunction after acute myocardial infarction: prevalence, clinical characteristics, and prognostic importance. American Journal of Cardiology 2006;97:13F-25F.
